# Episodic Memories Among Irritable Bowel Syndrome (IBS) Patients: An Important Aspect of the IBS Symptom Experience

**DOI:** 10.3389/fpain.2022.892313

**Published:** 2022-06-16

**Authors:** Gregory S. Sayuk, Carol S. North, David E. Pollio, Britt M. Gott, David H. Alpers

**Affiliations:** ^1^Division of Gastroenterology, Washington University School of Medicine, St. Louis, MO, United States; ^2^Department of Psychiatry, Washington University School of Medicine, St. Louis, MO, United States; ^3^St. Louis Veterans Affairs Medical Center, St. Louis, MO, United States; ^4^The Altshuler Center for Education & Research, Metrocare Services, Dallas, TX, United States; ^5^Department of Psychiatry, The University of Texas Southwestern Medical Center, Dallas, TX, United States; ^6^Metrocare Services, Dallas, TX, United States

**Keywords:** irritable bowel syndrome, memory, abdominal pain, somatization, anxiety, depression

## Abstract

**Objective:**

Some IBS patients possess detailed memories of the events surrounding their bowel symptom onset (“episodic memories”). In this exploratory study we sought to: (1) examine memory relationship with gastrointestinal (GI) symptom severity, extraintestinal symptoms, and mood; (2) qualitatively explore memory valence and content in IBS patients with or without episodic memories.

**Methods:**

Referral IBS patients *n* = 29; age 47.0± 2.2 years, 79.3% female) enrolled in this cross-sectional, mixed methods research study. Participants completed validated specific memory instruments [Autobiographical Memory Test (AMT), Sentence Completion for Events from the Past Test (SCEPT)] and relevant questionnaires [IBS symptoms 10-cm visual analog scale); SF-36 Health-related quality of life (HRQOL); Perley-Guze and PHQ-15/12: somatization; Beck Depression/Anxiety Inventories). Qualitative analysis examined the content and valence of general memories.

**Results:**

14/29 (48.3%) of IBS subjects endorsed episodic memories of IBS symptom onset, often GI infections/enteritis (35.7%). Recall of the exact year (69%) and month (60%) of symptom onset were common. Episodic memories were associated with greater IBS symptom severity/bother, higher anxiety/depression, and poorer HRQOL. Though AMT and SCEPT memory specificity were not different based on episodic memories, overgeneralization to negatively-valenced cues in the AMT was associated with more severe IBS in those without episodic memory. Qualitative analysis revealed no observable differences in topic focus of IBS patients with and without episodic memories.

**Conclusions:**

IBS patients often endorse episodic memories associated with symptom onset, and this recall seems to associate with more severe symptoms. Overgeneralization responses to negative stimuli may lead to worse bowel symptoms in those without episodic memories. IBS memory specificity may associate with qualitative differences in processing psychosocial experiences and might be important to IBS pathophysiology.

## Introduction

Irritable bowel syndrome (IBS) is a heterogeneous gastrointestinal (GI) condition characterized by abdominal pain and associated alteration in stool frequency and consistency (1, 2). As a result of this recognized lack of uniformity in IBS, numerous attempts have been made to identify relevant IBS subgroups. Despite these efforts, no satisfactory subclassification has emerged to date. Most commonly, subclassification is defined by bowel pattern (constipation, diarrhea, mixed) though this has been useful mostly for developing pharmacotherapies targeted at alterations in intestinal motility and/or secretion (3). Moreover, these subgroups have never been fully validated clinically, in part because the classification can change during the course of the illness (4, 5) and in part because the definition of these subgroups does not reflect the fact that a distinctive bowel pattern, and likely is not the only important factor defining symptomatology in many IBS patients (6).

Other IBS subclassification schemes, such as grouping according to documentation of comorbid psychiatric diagnoses (7), or expression of current anxiety or depression symptoms (8, 9), also have been suggested. Although the association of psychiatric disorders and symptoms with IBS is well documented, clinical significance of this relationship remains unclear. While patients seen in psychiatric clinics often report symptoms that meet the diagnostic criteria for IBS, such bowel symptoms may not be of primary concern to the patient (10). At the same time, historical psychiatric disorders and current symptoms often accompany IBS when the latter disorder is the primary problem. In such cases, the psychiatric comorbidities presumably modify the response to IBS symptoms (9, 11). IBS patients with higher psychiatric burdens do endorse more severe symptoms and poorer health-related quality of life in referral populations (10).

During the clinical care of patients with IBS, two of the authors (GS and DHA) have noted that many patients provided histories of a specific event at the onset of their IBS symptoms. This observation raised the possibility that the response to, or memory of, symptom onset might have mechanistic relevance to the initial development, and propagation of, IBS symptoms in such individuals. Further, we hypothesized that the presence or absence of such “specific memories” might serve as an important, meaningful way of subgrouping IBS patients, and might be of phenotypic relevance (i.e., IBS symptom severity, non-GI. Indeed, memory specificity has demonstrated significance in mood disorders, such as depression, that often accompany IBS (12).

We proposed that exploring aspects of the response to, and recall of, IBS symptoms (i.e., the “experience of illness”) might be relevant to the heterogeneity of IBS presentations. Moreover, such an approach may eventually provide clues regarding the pathogenesis, clinical course, and perhaps even the optimal treatment of the IBS patient. Thus, we conducted an exploratory study to measure and stratify IBS patients for the presence or absence of a specific disease-initiating memory, with the intention of comparing the groups in terms of IBS symptom measures, as well as quantitative and qualitative responses to validated autobiographical memory questionnaires and interviews.

## Methods

### Subjects and Clinical Characteristics

Adult IBS patients (≥18 years old) presenting for care at a tertiary outpatient gastroenterology practice of one of the investigators were invited to participate in this cross-sectional study between March 1, 2014 and December 31, 2014. The study subjects completed comprehensive symptom questionnaires, assessing GI symptoms, non-GI somatization symptoms, mood, and health-related quality of life, including the: (1) Rome III Research Diagnostic Questionnaire (13), (2) Beck Depression Inventory (BDI), (3) Beck Anxiety Inventory (BAI), (4) Patient Health Questionnaire (PHQ15) somatization scale, and (5) SF-36 HRQOL index (detailed below). Rome III criteria were applied to confirm the clinical features of IBS in this patient group, as only subjects satisfying these IBS criteria were included in the interview phase of the study. Baseline demographic characteristics, including sex and age, were recorded for all participants. Written informed consent was obtained from all study participants prior to collection of medical history or clinical data. Study protocol approval by the Washington University School of Medicine Human Research Protection Office (Institutional Review Board) was obtained prior to study initiation.

### Study Instruments

#### Bowel Symptoms

GI symptom severity and bother were assessed using 10-cm visual analog scales (VAS), as previously described (10). Specifically, the questions queried: “When you have your gastrointestinal symptoms, how severe are they?” (0 = “not severe at all”, 10 = “extremely severe”) and “How much have you been bothered overall by your gastrointestinal symptoms over the preceding 2 weeks?” (0 = “not bothered at all”, 10 = “extremely bothered”). Previously these VAS symptom severity scores were demonstrated to correlate in a statistically significant manner with disease-specific instruments such as the Gastrointestinal Symptom Rating Scale-IBS (GSRS-IBS) (Pearson r = 0.52, *p* < 0.0001) (14). Frequency of GI symptoms (e.g., number of symptomatic days) in the previous 2 weeks also was recorded (from 0 to 14 days).

#### Psychological and Somatization Assessments

The Beck Depression Inventory (BDI) and Beck Anxiety Inventory (BAI) were used to evaluate the presence of recent mood symptoms. Each instrument is a self-reported 21-question, multiple-choice inventory validated to assess characteristic attitudes and symptoms of depression and anxiety, respectively (15–17). Although these depression and anxiety inventories are intended to detect current symptom burden and alone are insufficient to make psychiatric diagnoses, they do indeed correlate with lifetime diagnoses (18, 19).

The Patient Health Questionnaire (PHQ-15) is a self-reported inventory validated to assess for somatic symptom severity, with higher PHQ-15 scores approximating somatization (somatic symptom) disorder diagnoses (20). The PHQ-12 measures non-GI somatic symptoms in functional GI disorder (FGID) patients, excluding the PHQ-15 GI symptoms (“stomach pain”, “constipation, loose bowels, or diarrhea”, and “nausea, gas, or indigestion”) (10).

The Perley-Guze criteria were utilized as well-validated, comprehensive criteria for the diagnosis of somatization disorder (21–23), though Perley-Guze criteria do not translate into a DSM-5 diagnosis of somatic symptom disorder. The Perley-Guze symptom checklist assesses 59 different symptoms across 10 possible symptom groups. Only symptoms that are clinically relevant (result in “a lot of trouble”) and are without a medical explanation are counted as Perley-Guze symptoms. A Perley-Guze diagnosis of somatization requires the presence of ≥ 25 symptoms distributed among ≥ 9 of 10 possible symptom categories (20).

#### Health-Related Quality of Life (HRQOL)

The SF-36 is a validated assessment of health-related wellbeing and functional status. SF-36 measures HRQOL as an overall (global) score, and further can be subdivided into physical and mental component summary scores, gauging the influence of the individual's medical conditions on physical and emotional wellbeing and limitation of day-to-day and pleasurable activities (24). The SF-36 measures HRQOL along a scale of 0 to 100, with higher scores reflecting better quality of life; it is one of the most widely used generic health outcome assessment measures, and has been applied to the study of a multitude of diseases and conditions (25).

#### Memory Assessments

IBS subjects all were interviewed by a single physician investigator (AS) who queried them about their recollection of GI specific memories. The interviews were recorded and transcribed by a third party not involved in the research study. They were asked, “Do you have a recollection of a specific event occurring at the time of your symptoms onset?” This question was recorded in a yes/no fashion, and served as the basis for dichotomizing the IBS study subjects into those with and without GI specific memories. Furthermore, subjects were asked to recall “What year did your symptoms begin?” and in the event that they were able to recall the year, a follow-up question: “Do you remember what month your symptoms began?”. Those subjects with a GI specific memory also were asked about “What specific details you can recall about the event?” surrounding the onset of their IBS symptoms.

The second portion of the interview involved the Autobiographical Memory Test (AMT) (26), asking participants to produce a specific memory in response to a cue word presented by the interviewer within a 60-s time limit. The 10 cue words had clear emotional valence, including five positive words (i.e., happy, safe, interested, successful, surprised) and five negative words (i.e., sorry, angry, clumsy, hurt, lonely) (27).

An additional method of assessment of memory specificity was the Sentence Completion for Events from the Past Test (SCEPT), an 11-item instrument which provides sentence stems, and instructs the participant to “complete each of the sentences” (28). A sample item is “I still remember well how…”. In contrast to AMT, the SCEPT stems all are valence neutral.

The AMT and SCEPT further were coded into described, validated categories by one of the investigators (BG) not involved in the patient care, and blinded to the clinical data. These responses were designated as one of the six following pre-defined categories: (1) *specific memory*, “referring to one specific moment or a particular time, e.g. ‘How sad I was the day my grandfather died.”’; (2) *categoric memory*, “a repeated activity or a category of events without the specification of a particular time, e.g., ‘My grandmother used to play games with me when I was little.”’; (3) *extended memory*, “refers to an extended period of time which lasted more than one day, e.g., ‘I was ill for two weeks in a row last year,”’; (4) *semantic associate*, “referring to personal overgeneral semantic information, e.g., ‘I used to be a very shy girl.”’; (5) *omission*, in the event that the subject could either not generate a response or “did not seriously try to complete the response in a meaningful way”. Individuals' responses were then dichotomized as “specific memories” (category 1) or “overgeneral memories” (categories 2–5) (28).

Non-psychotic formal thought disorder (NFTD or NPFTD) was assessed in 27 categories of thought disorder (e.g., “vague”, “flippant”, “circumstantial”) by coding transcribed patient narratives from assessment of the AMT, and SCEPT. All of the NFTD ratings were performed by a single expert physician (CN) blinded to group assignment (11, 29).

### Statistical Analysis

#### Quantitative Methods

This study was analyzed with mixed methods approach. Grouped values were reported as mean ± standard error of mean for continuous variables, or median and range values for categorical variables. Between-group comparisons were performed using Student's t-tests, and Chi-square analyses were performed for binomial data as appropriate. In each case, *p* < 0.05 was required for statistical significance, with an assumption of unequal variances. In order to explore the relationship between specificity of memory and the clinical presentation of the IBS and related non-GI (somatization) symptoms, correlational analyses were performed with generation of Pearson correlation coefficients. Given the exploratory nature of this study, no adjustments for multiple comparisons were implemented. All of the reported statistical analysis was carried out using SPSS Statistics version 27 (IBM SPSS, Armonk, NY, USA and SAS version 9.4 (SAS Institute, Cary, NC, USA).

#### Qualitative Methods

Qualitative data on memory of onset of IBS, and autobiographical memories in relation to 5 positively and 5 negatively valenced emotions and memories of past events were collected in an audio-recorded interview by a physician on the investigative team. These interviews were transcribed and the collective content from all of the patients was subjected to qualitative analysis. To begin the qualitative analysis, one researcher (GS) read all of the interview transcripts and identified a total of 30 categories covering material from the AMT and SCEPT interviews. These 30 categories were then conceptually collapsed into four main themes: (1) Vocation including work, school, money, and material possessions; (2) Home Life and Relationships, including family, friends, relationships, love, childhood, home, and pets; (3) Health and Self, including medical and psychological content, self, and medications/drugs; and (4) content External to Self, including life events, politics, and entertainment. The number of responses to the AMT and SCEPT items were coded into the various themes and tabulated across the themes for each interview. Interrater reliability was calculated for SCEPT rating validity check for the instrument and was determined to be very high, with Kappa = 0.913 95% CI 0.846–0.980); the remainder of the coding was examined collaboratively by the two qualitative coders (GS and BG). Following initial coding, statistically significant results from the quantitative analysis were used to guide an examination of qualitative content. The counts of items coded in themes were compared with quantitative variables collected in this study. Both the counts of items in themes and the qualitative content within the themes were examined for differences between IBS groups with and without specific memories of their IBS onset.

## Results

### Quantitative Findings

#### Subject Demographics and Clinical Characteristics

A total of 29 IBS subjects (mean age 47.0 ± 2.2 years, 79.3% female) agreed to participate in the study and completed the baseline questionnaires and memory interview. Twenty (69.0%) of the participants recalled the year of onset of their symptoms, and 12/20 (60.0%) of these individuals recalled the specific month of their symptom development. Of the overall IBS subject population, 14 (48.3%) endorsed a memory of a specific “disease initiating” event in association with the onset of their IBS symptoms. The most common experiences recalled at the time of IBS symptom onset included symptoms consistent with infection (5/14, 35.7%), psychological stress, and a recent surgery (4/14, 28.6% for each, respectively).

In comparing the subjects reporting a specific memory in association with their IBS symptom onset, there were no differences demographically (Table 1). However, subjects with a specific disease initiating memory did report greater IBS symptom bother and endorsed more severe symptoms. This same cohort also was found statistically to have significantly higher anxiety scores (BAI) and trends toward greater recent depression symptoms (BDI). Further, the specific memory participants had higher recent non-GI somatoform symptoms on the PHQ-12, but did not have any detectable difference in medically unexplained symptoms or symptom groups on the Perley-Guze assessment. IBS patients with a specific memory trended toward poorer overall HRQOL, as well as statistical significance for lower physical and mental HRQOL.

**Table 1:** Baseline demographic and clinical characteristics.

Variable^*^	**All IBS subjects (*n* = 29)**	**No specific memory (*N* = 15)**	**Specific memory (*N* = 14)**	***P* value**
Mean age (years)	47.0 ± 2.2	44.4 ± 3.9	50.4 ± 3.2	0.18
Female gender (%)	23 (79.3%)	13 (86.7%)	10 (71.4%)	0.31
IBS Symptom severity (VAS)	6.6 ± 0.6	5.8 ± 0.8	7.8 ± 0.4	0.057
IBS Symptom bother (VAS)	6.5 ± 0.6	5.6 ± 0.8	7.9 ± 0.4	0.029
IBS Symptom Frequency (days in last 2 weeks)^**^	10 (1,14)	10 (1,14)	10 (2,12)	0.83
Beck Depression Inventory (BDI)	13.2 ± 1.5	11.2 ± 1.9	16.4 ± 2.1	0.08
Beck Anxiety Inventory (BAI)	12.2 ± 1.5	9.6 ± 1.3	16.2 ± 2.8	0.028
PHQ-15	12.3 ± 0.8	11.0 ± 1.0	16.4 ± 2.1	0.012
PHQ-12	7.8 ± 0.7	6.8 ± 0.9	9.4 ± 0.6	0.026
Perley-Guze symptoms	10.4 ± 1.1	10.6 ± 1.7	10.1 ± 1.4	0.84
Perley-Guze symptom groups	5.3 ± 0.5	5.4 ± 0.7	5.2 ± 0.6	0.82
SF-36 total	53.8 ± 5.0	60.9 ± 6.2	42.6 ± 6.4	0.059
SF-36 physical	48.3 ± 5.5	57.1 ± 6.5	34.6 ± 7.6	0.041
SF-36 mental	54.9 ± 4.7	59.4 ± 6.4	47.9 ± 6.1	0.021

* * Mean ± standard error of mean (SEM) unless otherwise noted*.

** *Median (range)*.

#### Memory Assessments

Overall, the Autobiographical Memory Test revealed specific memories in 16.6% of the responses across all the IBS subjects (conversely overgeneral memories are documented in 83.4% of cases) (Table 2). There were no significant differences in AMT specific memories, either overall, or with regard to cue word valence in the two IBS disease initiating memory subgroups.

**Table 2:** Autobiographical memory test and sentence completion for events from the past test (SCEPT) by IBS subgroups.

**Variable**	**All IBS subjects (*n* = 29)**	**IBS-No specific memory (*N* = 15)**	**IBS-Specific memory (*N* = 14)**	***P* value**
**Autobiographical Memory Test (AMT)**				
Specific memory-total	48 (16.6%)	25 (16.7%)	23 (16.4%)	0.96
Overgeneral memory-total	242 (83.4%)	125 (83.3%)	117 (83.6%)	
Specific memory-positive	22 (15.2%)	9 (12.0%)	13 (18.6%)	0.27
Overgeneral memory-positive	123 (84.8%)	66 (88.0%)	57 (81.4%)	
Specific memory-negative	26 (17.9%)	16 (21.3%)	10 (14.3%)	0.27
Overgeneral memory-negative	119 (82.1%)	59 (78.7%)	60 (85.7%)	
**Sentence Completion for Events from the Past (SCEPT)**				
Specific memory	91 (31.8%)	43 (30.0%)	48 (33.6%)	0.53
Overgeneral memory	195 (68.2%)	100 (70.0%)	95 (66.4%)	

*Overgeneral memories, as recorded on the Autobiographical Memory Test (AMT) and Sentence Completion for Events from the Past Test (SCEPT) among the overall IBS cohort were quantified as described in the Methods. These overgeneral memories (total, and by positive/negative valence on the AMT) were further correlated with these same clinical measures*.

Twenty-six of the 29 (89.7%) subjects also participated in the Sentence Completion for Events from the Past Test (SCEPT). Here the overall rate of specific memories in the IBS population was higher at 31.8%. However, again there were no differences between the specific memory and no specific memory IBS subgroups.

#### Correlation of Specific Memories and Clinical Presentation

In order to explore the relationship between objective testing detection of specific memories on the AMT and SCEPT, correlation matrices were generated. In the AMT across the entire IBS study population, greater overgeneralization of memories suggested a relationship with more severe IBS symptoms and greater numbers of IBS symptomatic days, as well as higher scores on the PHQ-15 and PHQ-12, and worse HRQOL, particularly on the SF 36 mental (Table 3, line 1). These observations were driven particularly by the IBS subgroup with no specific memory at symptom onset (Table 3, line 3). The relationships observed were not particularly relevant in the AMT positive valence cues among any of the IBS subgroups or the overall study population (Table 3, line 4); rather, these effects appear to track most closely in the negative valence AMT cues. Statistically significant positive correlations in the overall IBS study population were observed with regard to IBS severity and symptomatic days as well as the PHQ-15 and PHQ-12, the BAI, and a negative correlation with SF-36 total and SF-36 mental supplement domains (Table 3, line 7). As with the correlations of overgeneralization itself, these correlations with negative valence cues were driven more by the IBS subgroup with no specific memory at symptom onset (Table 3, line 8). Examining the SCEPT, a positive correlation was only detected with regard to overgeneral responses in the total IBS population and symptoms on the Perley-Guze inventory (r = 0.412, *p* = 0.045). Again, this appeared to be predominantly driven by the IBS subgroups without recall of a specific memory related to symptom onset (r = 0.485, *p* = 0.11).

**Table 3:** Correlation of overgeneral memories with ibs patient clinical characteristics, mood, and health related quality of life.

	**# P-G**	**# P-G**	**P-G**,	**IBS Sx**	**IBS Sx**	**IBS Sx**			**BAI**	**BDI**	**SF36**	**SF36**	**SF36**
	**Sxs**	**groups**	**No GI Sx**	**severity**	**bother**	**days**	**PHQ15**	**PHQ12**	**total**	**total**	**total**	**physical**	**mental**
Total AMT Overgeneral, All IBS Subjects	0.192	0.130	0.196	0.478	0.295	0.456	0.415	0.429	0.377	0.002	−0.371	−0.182	−0.475
Total AMT Overgeneral, IBS-No Specific Memory	0.015	−0.085	0.131	0.716^*^	0.348	0.787^*^	0.596	0.495	0.596	0.203	−0.722^*^	−0.648	−0.71^*^
Total AMT Overgeneral, IBS-Specific Memory	0.366	0.316	0.244	0.125	0.400	−0.070	0.254	0.499	0.304	−0.304	0.065	0.392	−0.157
Positive Valence AMT Overgeneral, All IBS Subjects	0.064	−0.031	0.097	0.131	0.062	0.198	0.024	0.064	0.020	−0.260	0.005	0.187	−0.156
Positive Valence AMT Overgeneral, IBS-No Specific Memory	−0.096	−0.184	0.078	0.254	0.050	0.454	0.162	0.064	0.347	−0.093	−0.376	−0.304	−0.397
Positive Valence AMT Overgeneral, IBS-Specific Memory	0.163	0.050	0.100	0.292	0.544	−0.117	0.117	0.363	0.005	−0.354	0.238	0.529	−0.024
Negative Valence AMT Overgeneral, All IBS subjects	0.308	0.266	0.288	0.534^*^	0.418	0.571^*^	0.709^**^	0.673^**^	0.618^**^	0.261	−0.586^*^	−0.458	−0.576^*^
Negative Valence AMT Overgeneral, IBS-No Specific Memory	0.223	0.095	0.267	0.610	0.400	0.744^*^	0.741^*^	0.661^*^	0.603	0.265	−0.64^*^	−0.540	−0.624
Negative Valence AMT Overgeneral, IBS-Specific Memory	0.482	0.544^*^	0.335	−0.213	−0.015	0.040	0.380	0.520	0.686	−0.089	−0.258	−0.008	−0.319

*#P-G Sx, number of symptoms endorsed on Perley-Guze inventory; #P-G Groups, number of different Perley-Guze symptom groups; #P-G No GI Sx, number of symptoms endorsed on Perley-Guze inventory, excluding GI symptoms*.

*Sx, symptoms; PHQ, Patient Health Questionnaire; BAI, Beck Anxiety Inventory; BDI, Beck Depression Inventory; SF-36, Short Form 36 Health related quality of life*.

*Pearson correlation coefficients reported*.

* *P < 0.05*;

** *P < 0.01*.

### Qualitative Findings

Although the quantitative responses did not separate the groups with and without a disease-initiating memory, qualitative differences between these two groups were observed in two of the four themes (Home Life and Relationships and content External-to-Self). For the IBS group without a disease-initiating memory, somatoform characteristics were statistically significantly associated with the amount of specific topical memory content generated for the two themes. Greater content in the AMT Home Life and Relationships theme was positively associated with somatoform characteristics (level of NFTD: r = 0.55, *p* = 0.034; number of functional disorders: r = 0.61, *p* = 0.016). Greater content in the SCEPT External to Self-theme was negatively associated with somatoform characteristics (number of somatoform symptoms: r = −0.90, *p* < 0.001; number of positive somatoform symptom groups: r = −0.66, *p* = 0.028). In contrast, focused examination of the IBS group with a disease-initiating memory found that specific topical memory content was not positively associated with number of somatoform symptoms or symptom groups. Additionally, the positive association of AMT Home Life and Relationships with the number of functional disorders in the group without a disease-initiating memory was statistically significant in the opposite direction to the association with SCEPT Home Life and Relationships (r = −0.62, *p* = 0.040) in the group with a disease- initiating memory.

The two themes with associations between somatoform characteristics and amounts of content in specific themes were examined for their volume and content in the IBS groups with and without a disease-initiating memory. The amount of total content in the AMT Home Life and Relationships theme was greater in the memory group (2,137 words) than in the non-memory group (1,476 words). For this theme, the content of the responses in the two groups, while different in amount of detail, was relatively similar in terms of focus. For example, content focused on both positive and negative events. In terms of positive events, many individuals in both groups focused on pregnancy and childbirth: for example, “Just having heard I was going to have a baby was a surprise.” (memory) and “the day my daughter was born was the first best day of my life!” (no memory). In terms of negative events, individuals in both groups focused on relationship-focused events, such as divorce—“When I divorced my children's mother was the worst period of my life.” (memory), infidelity— “When my cousin had sex with my husband, I was angry.” (no memory), and death— “I am still hurting inside for not being able to talk to my mom and dad since their death.” (memory), “I will never forget the day we buried my grandmother” (no memory). Additionally, this theme had similar negative and positive valence responses (33% positive and 48% negative in the no disease-initiating memory group and 22% positive and 49% negative in the disease-initiating memory group).

For the External-to-Self theme, the two memory groups were similar in content and detail, with the most frequent responses focusing on past travel, both generically “In the past I used to do a lot of world traveling to great and unusual destinations” (no memory) and to a number of specific locations, including Branson, Missouri, Las Vegas, and Los Angeles (both groups). Individuals also mentioned more complex events, such as moving: “Last year we moved to St. Louis” (memory).

## Discussion

In this study, we identified that nearly one-half of IBS patients in a tertiary gastroenterology referral center recall a specific event in close association with the onset of their IBS symptoms. Some potentially important clinical differences were found between IBS subjects endorsing specific memories of such events, when compared to patients without such memories. In particular, patients with specific disease-associated memories had more severe and bothersome IBS symptoms as compared to their counterparts with no such memory. IBS patients with specific memories also scored higher on measures of mood severity (anxiety more so than depression) and somatoform symptoms, and endorsed overall lower health related quality of life. In examining validated measures of memory specificity, overgeneral responses on the Autobiographical Memory Test positively correlated with symptoms of IBS, both severity and bother, as well as poor health related quality of life. These observations appeared to be driven by the negative valence cues of the AMT, especially among the IBS patients with no specific memory. Thus, although there were no observable differences in numeric representation of the topics discussed by the IBS patients with and without specific memories of their disease onset, content provided by those with specific memories was positively associated with somatization and functional disorders for home. These findings suggest differences in IBS patients with and without specific memories of disease onset in how they process their psychosocial experiences of relationships and aspects of life external to themselves in relation to their IBS.

Autobiographical memory and memory specificity has been a topic of considerable interest in the psychiatric literature for several decades. However, it remains an area of controversy, with some studies failing to demonstrate a relationship between memory specificity and recent mood (30, 31) while others have suggested that depressive disorders may moderate the individual's memory specificity on autobiographical testing (32, 33). Interestingly, this compromise of memory specificity also has been described in association following traumatic events and posttraumatic stress disorder (34, 35), with abuse and trauma experiences also commonly reported in IBS patient populations (36, 37). These studies have not been designed to evaluate the contribution of the entirety of comorbidities that may associate with mood disorders, and to our knowledge there have been no studies to date that have evaluated patient cohorts established on the basis of medically unexplained, physical symptoms such as in IBS, a disorder frequently found in association with depression and/or anxiety. From a mechanistic perspective, it has been suggested that memory overgeneralization may result from the interaction of a ruminative focus on one's self, impairments in executive control, and a functional avoidance of memory retrieval of aversive events as a protective mechanism that results in distress reduction (12, 38, 39). With regard to mood, overgeneral memories have been suggested to predict the clinical course of the depressed patient, serving as a marker of treatment refractoriness and suggested to be a trait marker for persistent depression (40).

The individual responses of IBS patients to their symptoms of IBS likely has relevance to their symptom experience and healthcare utilization 6, though such responses have not been systematically studied. Attempts to account for patients' responses to their symptoms have been made by examining non-GI symptoms and healthcare utilization in addition to the GI symptoms, but such efforts have not resulted in definable subgroups of IBS patients (2, 6). Biomarkers also have been used in attempts to identify patients with the presumed underlying pathophysiology of visceral hypersensitivity, but without clear results (41).

Illness behavior involves the response to illness by seeking treatment, and is shaped by culture and expectations about how to behave when ill also differ according to the patients' unique individual biographies (42). Thus, although “two individuals may have the same disease, no two illness experiences are the same” (43). There exists a large literature on the experience of illness, but that approach has not been widely applied to IBS; however, the concept seems quite relevant. For example, recent work suggested that IBS patients who develop severe food avoidance and restriction may constitute a subgroup with more severe symptoms and poorer HRQOL (44). It is essential that the physician recognize that hidden behind the façade of the disease and its diagnosis is a story, unique to individual patients and their responses to the disease, or “illness narrative”. From the current study, we discern that a substantial percentage of IBS patients regard a specific event, such as an infection, acute psychological stress, or a recent surgery as an important part of this illness narrative with similar content of the topics of their concerns, but with different emphasis of content related to relationships and experiences external to self in the two groups. Application of autobiographical memory testing on an exploratory basis suggests such memories might convey clinical relevance, including the experience of non-GI somatoform symptoms, particularly in an individual who expresses overgeneralization in response to negative valence.

Strengths of this study included the examination of a carefully phenotyped IBS patient population diagnosed by expert gastroenterologists and verified using established diagnostic criteria. Further, the evaluation of memory specificity in this study relied upon the prospective collection of established autobiographical memory instruments with scoring and interpretation in a blinded fashion. This study was not without limitations. Given the cross-sectional design of this investigation, comments on causality cannot be offered. Further, without a control population one cannot be certain that the observations from the study are specific to IBS. The memory recall, in some cases, dated back several years which could have influenced the accuracy and influence on current symptoms. Further, the study focus on a referral gastroenterology population limits somewhat the generalizability of the results of the current trial to community-based IBS populations at large Finally, our smaller sample size in this exploratory study limited our ability to conduct additional statistical analyses attempting to control for some of the comorbid mood factors as potential confounders in our results.

In summary, this exploratory study found that substantial percentage of IBS patients endorse memories of a specific event at the time of their symptom onset, a factor that may predict more severe IBS symptoms, and perhaps non-GI comorbidities and poor quality of life. Overgeneralization of memory, particularly in relation to negatively valenced terms, too may portend worse IBS symptoms in those without specific memories relating to initiation of their symptoms. These preliminary observations should inspire additional work to determine whether these findings extrapolate to a broader IBS population, and moreover to better understand the implications with regard to clinical course and therapeutic outcomes. Longer term, it is conceivable that these psychological factors could provide for the identification of clinically-relevant IBS subgroups that benefit from unique behavioral and pharmacotherapeutic approaches.

## Data Availability Statement

The raw data supporting the conclusions of this article will be made available by the authors, without undue reservation.

## Ethics Statement

The studies involving human participants were reviewed and approved by Washington University Human Research Protection Office. The patients/participants provided their written informed consent to participate in this study.

## Author Contributions

GS, DA, CN, and DP: study concept and design, data analysis and interpretation, and manuscript authorship. BG: data extraction and analysis and manuscript authorship. All authors contributed to the article and approved the submitted version.

## Conflict of Interest

The authors declare that the research was conducted in the absence of any commercial or financial relationships that could be construed as a potential conflict of interest.

## Publisher's Note

All claims expressed in this article are solely those of the authors and do not necessarily represent those of their affiliated organizations, or those of the publisher, the editors and the reviewers. Any product that may be evaluated in this article, or claim that may be made by its manufacturer, is not guaranteed or endorsed by the publisher.

## References

[1] Mearin F Lacy BE Chang L Chey WD Lembo AJ Simren M Spiller R. Bowel disorders. Gastroenterology. (2016) S0016-5085(16)00222-5. 10.1053/j.gastro.2016.02.031 27144627

[2] Su AM Shih W Presson AP Chang L. Characterization of symptoms in irritable bowel syndrome with mixed bowel habit pattern. Neurogastroenterol Motil. (2014) 26:36–45. 10.1111/nmo.12220 23991913 PMC3865067

[3] Lacy BE Patel NK. Rome criteria and a diagnostic approach to irritable bowel syndrome. J Clin Med. (2017) 6. 10.3390/jcm6110099 29072609 PMC5704116

[4] Barberio B Houghton LA Yiannakou Y Savarino EV Black CJ Ford AC. Symptom stability in Rome IV vs Rome III irritable bowel syndrome. Am J Gastroenterol. (2021) 116:362–71. 10.14309/ajg.0000000000000946 33009062

[5] Garrigues V Mearin F Badia X Balboa A Benavent J Caballero A. Change over time of bowel habit in irritable bowel syndrome: a prospective, observational, 1-year follow-up study (RITMO study). Aliment Pharmacol Ther. (2007) 25:323–32. 10.1111/j.1365-2036.2006.03197.x 17217445

[6] Polster AV Palsson OS Tornblom H Ohman L Sperber AD Whitehead WE. Subgroups of IBS patients are characterized by specific, reproducible profiles of GI and non-GI symptoms and report differences in healthcare utilization: a population-based study. Neurogastroenterol Motil. (2019) 31:e13483. 10.1111/nmo.13483 30393924

[7] Young SJ Alpers DH Norland CC Woodruff RA. Jr., Psychiatric illness and the irritable bowel syndrome. Practical implications for the primary physician. Gastroenterology. 70:162–6. 10.1016/S0016-5085(76)80002-9 1248677

[8] Hausteiner-Wiehle C Henningsen P. Irritable bowel syndrome: relations with functional, mental, somatoform disorders. World J Gastroenterol. (2014) 20:6024–30. 10.3748/wjg.v20.i20.6024 24876725 PMC4033442

[9] Muscatello MR Bruno A Mento C Pandolfo G Zoccali RA. Personality traits and emotional patterns in irritable bowel syndrome. World J Gastroenterol. (2016) 22:6402–15. 10.3748/wjg.v22.i28.6402 27605876 PMC4968122

[10] Vu J Kushnir V Cassell B Gyawali CP Sayuk GS. The impact of psychiatric and extraintestinal comorbidity on quality of life and bowel symptom burden in functional GI disorders. Neurogastroenterol Motil. (2014) 26:1323–32. 10.1111/nmo.12396 25070610

[11] North CS Hong BA Alpers DH. Relationship of functional gastrointestinal disorders and psychiatric disorders: implications for treatment. World J Gastroenterol. (2007) 13:2020–7. 10.3748/wjg.v13.i14.2020 17465442 PMC4319119

[12] Williams JM Barnhofer T Crane C Herman D Raes F Watkins E. Autobiographical memory specificity and emotional disorder. Psychol Bull. (2007) 133:122–48. 10.1037/0033-2909.133.1.122 17201573 PMC2834574

[13] Drossman DA Delvaux M Spiller RC Talley NJ Thompson WG Whitehead W. Rome III: The Functional Gastrointestinal Disorders, Degnon Associates, Inc. New York, NY: Basic Books (2006).

[14] Kushnir VM Cassell B Gyawali CP Newberry RD Kibe P Nix BD. Genetic variation in the beta-2 adrenergic receptor (ADRB2) predicts functional gastrointestinal diagnoses and poorer health-related quality of life. Aliment Pharmacol Ther. (2013) 38:313–23. 10.1111/apt.12378 23786226 PMC4017784

[15] Beck AT Epstein N Brown G Steer RA. An inventory for measuring clinical anxiety: psychometric properties. J Consult Clin Psychol. (1988) 56:893–7. 10.1037/0022-006X.56.6.893 3204199

[16] Beck AT Ward CH Mendelson M Mock J Erbaugh J. An inventory for measuring depression. Arch Gen Psychiatry. (1961) 4:561. 10.1001/archpsyc.1961.01710120031004 13688369

[17] Steer RA Ball R Ranieri WF Beck AT. Dimensions of the beck depression inventory-II in clinically depressed outpatients. J Clin Psychol. (1999) 55:117–28. 10.1002/(SICI)1097-4679(199901)55:1<117::AID-JCLP12>3.0.CO;2-A 10100838

[18] Subica AM Fowler JC Elhai JD Frueh BC Sharp C Kelly EL. Factor structure and diagnostic validity of the Beck Depression Inventory-II with adult clinical inpatients: comparison to a gold-standard diagnostic interview. Psychol Assess. (2014) 26:1106–15. 10.1037/a0036998 24932646

[19] Carrington EV Heinrich H Knowles CH Fox M Rao S Altomare DF. The international anorectal physiology working group (IAPWG) recommendations: Standardized testing protocol and the London classification for disorders of anorectal function. Neurogastroenterol Motil. (2020) 32:e13679. 10.1111/nmo.13679 31407463 PMC6923590

[20] Kroenke K Spitzer RL Williams JB. The PHQ-15: validity of a new measure for evaluating the severity of somatic symptoms. Psychosom Med. (2002) 64:258–66. 10.1097/00006842-200203000-00008 11914441

[21] Feighner JP Robins E Guze SB Woodruff RA Winokur G Munoz R. Diagnostic criteria for use in psychiatric research. Arch Gen Psychiatry. (1972) 26:57–63. 10.1001/archpsyc.1972.01750190059011 5009428

[22] Perley MJ Guze SB. Hysteria–the stability and usefulness of clinical criteria. A quantitative study based on a follow-up period of six to eight years in 39 patients. New Eng J Med. (1962) 266:421–6. 10.1056/NEJM196203012660901 14485393

[23] North CS Yutzy SH. Somatization Disorder, Goodman and Guze's Psychiatric Diagnosis, 7th Ed., Oxford: Oxford Univeristy Press. (2018) p. 207–26. 10.1093/med/9780190215460.003.0008

[24] Winslow E Clouse R Desai K Frisella P Gunsberger T Soper N. Influence of spastic motor disorders of the esophageal body on outcomes from laparoscopic antireflux surgery. Surg Endosc. (2003) 17:738–45. 10.1007/s00464-002-8538-y 12618949

[25] McHorney CA Ware JE Jr., Lu JF, Sherbourne CD. The MOS 36-item Short-Form Health Survey (SF-36): III. Tests of data quality, scaling assumptions, and reliability across diverse patient groups. Medical Care. (1994) 32:40–66. 10.1097/00005650-199401000-00004 8277801

[26] Williams JM Broadbent K. Autobiographical memory in suicide attempters. J Abnorm Psychol. (1986) 95:144–9. 10.1037/0021-843X.95.2.144 3711438

[27] Van Vreeswijk MF Wilde EJDe. Autobiographical memory specificity, psychopathology, depressed mood and the use of the Autobiographical Memory Test: a meta-analysis. Behav Res Ther. (2004) 42:731–43. 10.1016/S0005-7967(03)00194-3 15081887

[28] Raes F Hermans D Williams JM Eelen P. A sentence completion procedure as an alternative to the Autobiographical Memory Test for assessing overgeneral memory in non-clinical populations. Memory. (2007) 15:495–507. 10.1080/09658210701390982 17613793 PMC2796567

[29] North CS Hansen K Wetzel RD Compton W Napier M Spitznagel EL. Nonpsychotic thought disorder: objective clinical identification of somatization and antisocial personality in language patterns. Compr Psychiatry. (1997) 38:171–8. 10.1016/S0010-440X(97)90071-7 9154374

[30] Harvey AG Bryant RA Dang ST. Autobiographical memory in acute stress disorder. J Consult Clin Psychol. (1998) 66:500–6. 10.1037/0022-006X.66.3.500 9642888

[31] Scott J Stanton B Garland A Ferrier IN. Cognitive vulnerability in patients with bipolar disorder. Psychol Med. (2000) 30:467–72. 10.1017/S0033291799008879 10824667

[32] Wessel I Meeren M Peeters F Arntz A Merckelbach H. Correlates of autobiographical memory specificity: the role of depression, anxiety and childhood trauma. Behav Res Ther. (2001) 39:409–21. 10.1016/S0005-7967(00)00011-5 11280340

[33] Ridout N Dritschel B Matthews K O'Carroll R. Autobiographical memory specificity in response to verbal and pictorial cues in clinical depression. J Behav Ther Exp Psychiatry. (2016) 51:109–15. 10.1016/j.jbtep.2016.01.002 26808234

[34] Barry TJ Lenaert B Hermans D Raes F Griffith JW. Meta-analysis of the association between autobiographical memory specificity and exposure to trauma. J Trauma Stress. (2018) 31:35–46. 10.1002/jts.22263 29513912

[35] Brown AD Root JC Romano TA Chang LJ Bryant RA Hirst W. Overgeneralized autobiographical memory and future thinking in combat veterans with posttraumatic stress disorder. J Behav Ther Exp Psychiatry. (2013) 44:129–34. 10.1016/j.jbtep.2011.11.004 22200095

[36] Leserman J Drossman DA. Relationship of abuse history to functional gastrointestinal disorders and symptoms: some possible mediating mechanisms. Trauma Violence Abuse. (2007) 8:331–43. 10.1177/1524838007303240 17596349

[37] Ringel Y Drossman DA Leserman JL Suyenobu BY Wilber K Lin W. Effect of abuse history on pain reports and brain responses to aversive visceral stimulation: an FMRI study. Gastroenterology. (2008) 134:396–404. 10.1053/j.gastro.2007.11.011 18242208

[38] J.A. Sumner. The mechanisms underlying overgeneral autobiographical memory: an evaluative review of evidence for the CaR-FA-X model. Clin Psychol Rev. (2012) 32:34–48. 10.1016/j.cpr.2011.10.003 22142837 PMC3246105

[39] Sumner JA Mineka S Adam EK Craske MG Vrshek-Schallhorn S Wolitzky-Taylor K. Testing the CaR-FA-X model: investigating the mechanisms underlying reduced autobiographical memory specificity in individuals with and without a history of depression. J Abnorm Psychol. (2014) 123:471–86. 10.1037/a0037271 24978693 PMC4122646

[40] Brittlebank AD Scott J Williams JM Ferrier IN. Autobiographical memory in depression: state or trait marker? Br J Psychiatry. (1993) 162:118–21. 10.1192/bjp.162.1.118 8425125

[41] Sood R Law GR Ford AC. Diagnosis of IBS: symptoms, symptom-based criteria, biomarkers or 'psychomarkers'. Nat Rev Gastroenterol Hepatol. (2014) 11:683–91. 10.1038/nrgastro.2014.127 25069544

[42] Kleinman. The Illness Narratives: Suffering, Healing, and the Human Condition. New York, NY: Basic Books. (1988).

[43] Fleischman S. I am..., I have..., I suffer from...: A Linguist Reflects on the Language of Illness and Diseas. J Med Humanities. (1999) 20:3–32. 10.1023/A:1022918132461

[44] Melchior C Algera J Colomier E Tornblom H Simren M Storsrud S. Food avoidance and restriction in irritable bowel syndrome: relevance for symptoms, quality of life and nutrient intake. Clin Gastroenterol Hepatol. (2021) 116:732–60. 10.1016/S0016-5085(21)02164-8 34229035

