# Supplementary material for: Episodic Memories Among Irritable Bowel Syndrome (IBS) Patients: An Important Aspect of the IBS Symptom Experience
Source: Front Pain Res (Lausanne). 2022 Jun 16;3:892313. doi: 10.3389/fpain.2022.892313 (PMC9243497; doi:10.3389/fpain.2022.892313)
Supplement: Supplementary file 1 [file Table_1.pdf]

10.3389/fpain.2022.892313

Supplemental Table 1

<u>AMT and SCEPT Home Life and Relationships</u>

No disease-initiating memory group

Happy when my children were born.

The day my daughter was born was the first best day of my life!

My husband had an affair. It was very painful.

When my husband had an affair, it made me angry.

When my cousin had sex with my husband, I was angry.

I will never forget the day we buried my grandmother s.

Disease-initiating memory group

Just having heard I was going to have a baby was a surprise.

The most emotional hurt that I got was when my ex cheated on me with my girlfriend and got her pregnant.

I went through a very painful divorce.

When I divorced my children's mother was the worst period of my life.

I am still hurting inside for not being able to talk to my mom and dad since their death.

I will never forget the day we buried my grandmother.

<u>SCEPT External to Self</u>

No disease-initiating memory group

Last year we vacationed in the southwest states.

Last year my family spent our vacation at Branson, Missouri.

Last year I went to Vegas.

Disease-initiating memory group

Last year we moved to St. Louis.

Last year we went to Los Angeles, California.

In the past I used to do a lot of world traveling to great and unusual destinations
